# Differences in Anthropometric, Sleep and Respiratory Characteristics between Hypercapnic and Normocapnic Patients with COPD-OSA Overlap Syndrome

**DOI:** 10.3390/jpm14060600

**Published:** 2024-06-04

**Authors:** Athanasios Voulgaris, Kostas Archontogeorgis, Konstantina Chadia, Dimitra Siopi, Evangelia Nena, Paschalis Steiropoulos

**Affiliations:** 1 MSc Program in Sleep Medicine, Medical School, Democritus University of Thrace, 68100 Alexandroupolis, Greece; thanasisvoul@hotmail.com (A.V.); k.archontogeorgis@yahoo.it (K.A.); disiopi@gmail.com (D.S.); 2 Department of Pneumonology, Medical School, Democritus University of Thrace, 68100 Alexandroupolis, Greece; con_chadia@yahoo.gr; 3 Laboratory of Hygiene and Environmental Protection, Medical School, Democritus University of Thrace, 68100 Alexandroupolis, Greece; evangelianena@yahoo.gr

**Keywords:** chronic obstructive pulmonary disease, obstructive sleep apnea, overlap syndrome, hypercapnia, hypoventilation

## Abstract

Background: Overlap syndrome (OS), the coexistence of chronic obstructive pulmonary disease and obstructive sleep apnea, is frequently characterized by the presence of daytime hypercapnia (pCO_2_ ≥ 45 mmHg). The aim of this study was to investigate potential differences in anthropometric, sleep and respiratory characteristics between hypercapnic and normocapnic patients with OS. Methods: Consecutive patients who underwent polysomnography, pulmonary function testing and arterial blood gases and had been diagnosed with OS were enrolled in the study. Results: According to pCO_2_ levels in wakefulness, the patients were divided into group A, consisting of OS patients without hypercapnia (*n* = 108) or group B, consisting of OS patients with hypercapnia (*n* = 55). The majority of included patients in both groups were males (*n* = 92 in group A vs. *n* = 50 in group B). Group B had increased BMI (*p* = 0.001), neck (*p* = 0.017) and waist circumference (*p* = 0.013), higher scores in Epworth sleepiness scale (ESS) (*p* = 0.008), increased sleep efficiency (*p* = 0.033), oxygen desaturation index (*p* = 0.004) and time with oxyhemoglobin saturation <90% (*p* = 0.006) than group A. Also, Group B had decreased average and minimum oxyhemoglobin saturation during sleep (*p* < 0.001). Hypercapnic patients had lower FEV_1_% (*p* = 0.003), FVC% (*p* = 0.004), pO_2_ and pCO_2_ (*p* < 0.001 for both) values compared with normocapnic patients. In binary regression analysis, which assessed various predictors on the likelihood of having hypercapnia, it was found that BMI (OR: 1.313, 95% CI: 1.048–1.646, *p* = 0.018) and FVC (OR: 0.913, 95% CI: 0.845–0.986, *p* = 0.020) were the major determinants of hypercapnia in OS patients. Conclusions: Hypercapnic OS patients were more obese and sleepy and presented worse respiratory function in wakefulness and sleep hypoxia characteristics compared with normocapnic OS patients.

## 1. Introduction

Sleep has a significant impact on breathing and gas exchange even in normal individuals [1]. Its effects on pulmonary ventilation is even more pronounced in patients with chronic obstructive pulmonary disease (COPD) [2]. The coexistence of obstructive sleep apnea (OSA) in patients with COPD places an additional burden on patients affected by both diseases, a condition otherwise known as overlap syndrome (OS) [2,3].

In clinical practice, both OSA and COPD are among the most common chronic respiratory diseases. Recent data estimate the global prevalence of OSA and COPD at around one billion and 400 million people, respectively [4,5]. According to a recent systematic review, the prevalence of OS in the general population is low, ranging from 1 to 3.6% [6]. Nevertheless, in COPD populations, the prevalence of coexisting OSA ranged from 56.45% to 78% [7]. Similarly, studies including OSA patients revealed a diagnosis of COPD between 11.9 and 23.2% [7].

COPD and OSA are associated with a range of overlapping pathophysiological disturbances, which lead to systemic inflammation and oxidative stress [2], with common risk factors [8]. With regards to pathophysiology, COPD and OSA are frequently associated with hypoventilation [2]. The presence of repetitive apneas and hypopneas during sleep leads to intermittent nocturnal hypoxia and hypercapnia in patients with OSA [9]. In COPD, due to the expected loss of accessory muscle contraction combined with the impaired diaphragmatic function from hyperinflation, hypoventilation may initially appear during rapid eye movement (REM) sleep [10]. As the disease progresses, hypoventilation ensues during NREM sleep, and then diurnal hypoventilation is established due to air trapping, worsening hyperinflation and respiratory muscle fatigue [11].

Daytime hypercapnia is more pronounced in patients with OS when compared with patients with COPD or OSA alone [3,12]. Indeed, hypercapnia is highly prevalent among patients with OS, even in the presence of mild obstruction, indicating its multifactorial nature [12]. The higher burden of hypercapnia may be responsible for the excess mortality in patients with OS [13,14]. Therefore, the identification of hypercapnia in OS is of great importance highlighting the need for further studies to understand its relevance to patient outcomes.

So far, an earlier study did not reveal any significant differences between hypercapnic versus normocapnic patients with OS [12]. Moreover, it found that the contribution of overweight and reduced respiratory function could explain the hypercapnia in this patient group [12]. Most studies included patients with OSA or COPD compared with OS [15,16], but they did not assess if hypercapnic patients with OS had different characteristics compared to patients with OS and normocapnia. Therefore, the aim of the study was to investigate any differences in terms of anthropometric, sleep and respiratory characteristics between hypercapnic and normocapnic patients with OS and to examine the determinants of hypercapnia in OS.

## 2. Materials and Methods

### 2.1. Participants

This retrospective study was conducted at the sleep laboratory of the University General Hospital of Alexandroupolis, Greece. The study protocol (IRB: 14-1/27.01.2017) was approved by the institutional ethics committee and all procedures were conducted in accordance with the Helsinki Declaration of Human Rights [17]. Written informed consent was obtained from all participants.

Consecutive patients, who were referred to the sleep laboratory from 2011 to 2018 due to symptoms suggestive of sleep disordered breathing, were retrospectively evaluated for the study. Included patients fulfilled the following criteria: (a) newly diagnosed patients with COPD and OSA via spirometry and polysomnography respectively, (b) aged 40 years or older, (c) current or former tobacco smokers with at least ten pack-years of smoking history, and (d) complete patient data including arterial blood gases during wakefulness. Exclusion criteria were subjects with (a) exclusively central sleep apneas in polysomnography, (b) already use of positive airway pressure (PAP) or non-invasive ventilation (NIV), (c) recent history of COPD exacerbation or lower respiratory tract infection, (d) unstable cardiovascular comorbidity, (e) hypothyroidism or (f) any disease and drug related to hypercapnia.

### 2.2. Study Variables

A complete patient history was obtained from all participants, including the assessment for chronic respiratory symptoms and sleep habits, recording of comorbidities, current and previous medication use, tobacco exposure, as well as alcohol consumption. As former smoker was characterized anyone had quit smoking at the time of interview. Also, all patients were assessed for the presence of daytime sleepiness with the use of the validated Greek version of the Epworth Sleepiness Scale (ESS) [18]. The ESS is a self-administered questionnaire assessing the probability of falling asleep in a variety of daily circumstances (maximum score: 24; score > 10: excessive daytime sleepiness). The following clinical parameters were also recorded for all participants: anthropometric characteristics [height, weight, neck-hip-waist circumference, waist and hip circumference ratio, body mass index (BMI)], and a complete pulmonary and cardiac examination.

All patients underwent pulmonary function testing by spirometry (Chest Co., Tokyo, Japan) and analysis of arterial blood gases (ABGs) during wakefulness (ABL3000 autoanalyzer, Radiometer Co., Tokyo, Japan). ABGs were collected from the patient at rest from the radial artery of the patients the morning after polysomnography. Daytime hypercapnia was defined as carbon dioxide partial pressure (pCO_2_) ≥ 45 mmHg [19].

The diagnosis of COPD was based on the diagnostic criteria of the Global Initiative for Obstructive Lung Disease (GOLD) report. Briefly, the combination of clinically related symptoms, like dyspnea, cough, and/or exacerbations, in the setting of persistent airflow obstruction (post-bronchodilator forced expiratory volume in 1st second to forced volume capacity, FEV_1_/FVC ratio < 0.7) associated with a history of tobacco exposure or other noxious gases and particles established the diagnosis of COPD [20].

### 2.3. Polysomnography

All participants underwent an attended 8 h overnight polysomnography (PSG) from 22:00 to 06:00. (Alice^®^ 4, Philips Respironics, Murrysville, PA, USA). PSG included a standard montage of electroencephalogram, electro-oculogram, electromyogram and electrocardiogram channels. Monitoring of respiratory events was performed using combined oronasal thermistors and thoracic/abdominal strain gauges, while oxyhemoglobin saturation was assessed using a pulse oximeter placed on the index finger. Apneas, hypopneas and electroencephalogram recordings were manually scored according to guidelines [21]. Apnea was defined as a ≥90% of reduction in airflow for at least 10 s. Hypopnea was defined as a ≥30% reduction in airflow for at least 10 s in combination with oxyhemoglobin desaturation of at least 3% or an arousal registered by the electroencephalogram. The apnea–hypopnea index (AHI) was calculated from the average number of apneas and hypopneas per hour of sleep-recorded time. The oxygen desaturation index (ODI) was measured from the average number of oxyhemoglobin desaturation episodes per hour of sleep-recorded time. OSA was defined as an AHI ≥ 15 events/hour of sleep or as an AHI ≥ 5/h accompanied by OSA-related symptoms and/or comorbidities [21].

### 2.4. Statistical Analysis

The power of the study was calculated using G*Power software (version 3.1.9.7). Analysis for a two-tailed independent sample *t*-test for our study group’s samples yielded a power of 0.85 with an alpha of 0.05 and a medium effect size (d = 0.5). All analyses were carried out using IBM Statistical Package for Social Sciences (SPSS Inc. Released 2008. SPSS Statistics for Windows, Version 17.0. SPSS Inc., Chicago, IL, USA). Continuous variables were tested for normality of distribution with the Shapiro-Wilk test. All data are expressed as median (25th–75th percentile). The chi-squared test was used for the comparison of percentages between the groups. Correlations were analyzed with Pearson’s or Spearman’s correlation coefficient according to normality of distribution. Comparisons between means were studied with the student’s *t*-test or in case of skewed distribution, the non-parametric Mann–Whitney test. Independent predictors of hypercapnia between the two groups were identified using binary regression analysis. Statistical significance was defined at *p* < 0.05.

## 3. Results

A total of 163 consecutive patients (142 males), who were diagnosed with COPD and OSA, i.e., OS, from 2011 to 2018, at the sleep laboratory of the University General Hospital of Alexandroupolis, Greece, were retrospectively enrolled in the study. Based on the pCO_2_ levels, patients were divided into two groups: group A: OS patients without hypercapnia (*n* = 108) and group B: OS patients with hypercapnia (*n* = 55).

Most of included patients in both groups were males (*n* = 92 in group A vs. *n* = 50 in group B). The two groups did not differ in terms of gender (*p* = 0.302), age (*p* = 0.689) or smoking history (*p* = 0.357). Group B had higher BMI (35.2 Kg/m^2^ vs. 39.7 Kg/m^2^, *p* = 0.001), neck—(44 cm vs. 47 cm, *p* = 0.017) and waist—(124 cm vs. 132 cm, *p* = 0.013) circumference compared with group A. In Table 1, the anthropometric characteristics are displayed between the two groups.

With regards to sleep parameters, AHI was similar between the two groups’ patients (39.5 vs. 41 events/h of sleep, *p* = 0.403). Compared to group A, group B reported higher scores in ESS (9 for group A vs. 13 for group B, *p* = 0.008) and showed increased sleep efficiency (79.3% vs. 85.5%, *p* = 0.033) in PSG. Moreover, group B exhibited higher ODI (35.5 vs. 63 events/h of sleep, *p* = 0.004) and time with oxyhemoglobin saturation <90% (T < 90%, 20.7% vs. 38%, *p* = 0.006), as well as decreased average and minimum oxyhemoglobin saturation during sleep (91% vs. 89%, *p* < 0.001, 77% vs. 68%, *p* < 0.001, respectively).

Group B demonstrated worse respiratory function during wakefulness than group A. Specifically, OS patients with hypercapnia presented lower FEV_1_ (69.5% vs. 59%, *p* = 0.003) and FVC (74.5% vs. 63%, *p* = 0.004) compared to patients without hypercapnia. Furthermore, patients in group B exhibited lower oxygen partial pressure (pO_2_) (74 mmHg vs. 65 mmHg, *p* < 0.001) and higher pCO_2_ (41 mmHg vs. 49 mmHg, *p* < 0.001) compared to those in patients from group A. Comparisons of sleep parameters between the groups are presented in Table 2, while Table 3 displays the differences in respiratory function while awake.

A binary regression analysis was performed to assess the effect of various predictors on the likelihood of having hypercapnia. Namely, predictors included in the analysis were age, BMI, indices of respiratory function (FEV_1_, FVC) and sleep parameters (TST, sleep efficiency, AHI, arousal index, ODI, average and minimum oxyhemoglobin saturation and time spent with oxyhemoglobin saturation <90% during sleep). Among examined predictors, increased BMI (OR: 1.313, 95% CI: 1.048–1.646, *p* = 0.018) and decreased FVC (OR: 0.913, 95% CI: 0.845–0.986, *p* = 0.020) were the only determinants of hypercapnia in overlap patients.

## 4. Discussion

The present study tried to investigate the differences in clinical and laboratory characteristics between patients diagnosed with overlap syndrome with and without daytime hypercapnia. In our study, patients with OS and hypercapnia were more obese and reported higher levels of daytime sleepiness than those without hypercapnia. Moreover, this group of patients demonstrated worse nocturnal hypoxic parameters, more pronounced daytime hypoxemia and impaired lung function during wakefulness. Moreover, after adjustments for possible confounders, we found out that the determinants of daytime hypercapnia in this study were the BMI and the FVC.

Many patients with COPD, even with stable disease, experience subjective and objective sleep difficulties [22]. Furthermore, COPD is often accompanied by sleep disordered breathing, which is characterized by the presence of nocturnal hypoxemia and hypoventilation, as well as obstructive and central sleep apneas [22]. Indeed, presence of OSA is common in COPD patients, referred to as OS, and is even more prevalent in those patients with moderate to severe disease [23]. Overlap syndrome is associated with greater risk of nocturnal oxygen desaturation compared to patients with COPD or OSA, as a single diagnosis [24]. Moreover, patients with OS are at higher risk for developing daytime hypoxemia compared with patients with OSA or COPD [13,16]. Another aspect of pathophysiology during sleep is that patients with OS are more susceptible to nocturnal hypoventilation [25]. This type of sleep hypoventilation is characterized by nocturnal and diurnal hypercapnia of greater magnitude than either disease alone [25]. In our study, over one third of patients with OS had hypercapnia at the time of their initial evaluation. This confirms the literature that hypercapnia is more prevalent in OS compared to OSA and COPD.

Resta et al. assessed retrospectively the clinical and laboratory characteristics of 213 patients with COPD, OSA and OS [12]. The authors found that patients with OS had higher levels of pCO_2_ compared to those with COPD (44.59 vs. 39.63 mmHg; *p* < 0.005) and OSA (44.59 vs. 39.22 mmHg; *p* < 0.01) [12]. Interestingly, AHI levels were similar between patients with OS and OSA (40.46 vs. 41.59/h), but COPD patients exhibited greater severity of airflow obstruction compared with OS patients (FEV_1_% of predicted 47.31 vs. 62.93; *p* < 0.005) [12]. Similarly, a larger study demonstrated that OS patients had worse levels of awake pCO_2_ and pO_2_ compared with OSA patients, while no difference was observed between OS and COPD individuals in those levels [16]. ESS scores were higher in the OS and OSA groups than in COPD group, and T90% during sleep was higher in the OS as compared with the other two groups [16].

A more recent multicenter study, evaluating 509 OS and 1018 OSA patients from the European Sleep Apnoea Database (ESADA), revealed lower daytime pO_2_ levels and higher mean oxygen saturation during sleep in OS compared to OSA individuals, whereas no other differences were noted, including the levels of pCO_2_ [15]. Another study, comparing patients with OS and COPD only, found no difference in the levels of pCO_2_ [26]. Nevertheless, patients with OS were sleepier and demonstrated worse parameters of nocturnal oxygenation, but with higher FEV_1_ values than those in COPD patients [26].

Apart from an earlier study [12], the question remained open whether hypercapnic patients with OS exhibit worse sleep and respiratory characteristics than OS patients with normocapnia. Our study demonstrated that hypercapnic patients with OS had more time spent in light sleep (sleep stage N2) and exhibited higher scores in ESS and increased sleep efficiency than the patients with normocapnia. The latter was noted even though the groups did not differ in total sleep time and arousal index, reflecting more sleepiness in the group of patients with hypercapnia. A previous study evaluated a group of patients with OS and daytime hypercapnia or obesity hypoventilation syndrome [27]. The authors used quantitative EEG analyses and Delta/Alpha ratio as markers of EEG activation. In this study, the EEG spectral analysis showed a slower EEG spectral profile (i.e., reduced Delta/Alpha ratio of EEG), which was altered to an activated and faster EEG following improvements in ESS scores, nocturnal oxygenation parameters and pCO_2_ levels during wakefulness after treatment with PAP [27]. Moreover, in regression analysis, the change in pCO_2_ levels was the most significant predictor explaining both the variance in EEG and in ESS [27]. Taking these findings together, it seems that daytime hypercapnia is linked to distorted micro-sleep architecture, which is identified with more sophisticated metrics than the arousal index, and a higher degree of daytime sleepiness, as reported in our study.

Concerning respiratory parameters both in sleep and wakefulness, the study of Resta et al. [12] showed that hypercapnic patients with OS demonstrated non-significant but worse levels of FEV_1_ (56.5 vs. 66.5%), pO_2_ (65 vs. 73.61 mmHg) and T90% (59.11 vs. 42.64) compared with those in normocapnic patients with OS [12]. Contrary to these results, in our study, the group of patients with hypercapnia demonstrated greater airflow obstruction and daytime hypoxemia in comparison to the normocapnic group, regarding the COPD severity and respiration respectively. Also, they exhibited higher ODI and worse levels of T90% and average and minimum oxyhemoglobin saturation than those levels in the group of patients with normocapnia, even though both groups had similar AHI values. Previous studies on OSA populations reported a link between pCO_2_ levels and severity of nocturnal oxygenation, reflected in average and minimum oxygen saturation and TST < 90%, irrespective of AHI [28,29]. It is known that the severity of nocturnal oxygen desaturation is largely dependent on the degree of nocturnal and diurnal hypoventilation, in other words, the levels of nocturnal and daytime pCO_2_ [30].

Schreiber et al., in a retrospective analysis, found that OS patients started on NIV had an increased prevalence of hypercapnia compared with the patients under CPAP treatment. The hypercapnic group had reduced BMI values and presented lower levels of FEV_1_ and worse average oxyhemoglobin saturation and T90% during sleep compared with the normocapnic group on CPAP treatment. These results are in line with our findings, indicating that patients with hypercapnia demonstrate poorer respiratory function and oxygenation during wakefulness and sleep.

Hypercapnia is developed due to an imbalance between excessive load and inadequate unloading of the carbon dioxide (CO_2_). Obesity may impair upper airway mechanics and increase body oxygen consumption and CO_2_ production. These phenomena are translated to increased work of breathing and impaired ventilatory drive [31]. The combination of ventilatory impairment, excess CO_2_ production and reduced ventilatory drive predisposes obese individuals finally to diurnal hypoventilation [31]. Another factor is that obesity is related with reductions in functional residual capacity, expiratory reserve volume and lung compliance [31]. This will result in decreased lung volumes and notably FVC and total lung capacity [32]. Of note, the degree of airflow obstruction, as reflected by the FEV_1_, could not explain the presence of hypercapnia in OS in our study. This is further supported by the study by Resta et al., who showed that patients with OS and less severe obstruction in PFTs may exhibit also hypercapnia [12]. Interestingly, sleep hypoventilation in OS is also mainly attributed to an increased upper airway resistance, indicating the additive effects of OSA [33].

Possible factors explaining the presence of hypercapnia in patients with OS are also under investigation. In one study including patients with OSA and without COPD, daytime hypercapnia was evident in 11% of the studied group and increased alongside increasing BMI values [19]. Predictors of hypercapnia in the OSA group were pO_2_, BMI, FVC and FEV_1_. Similar findings were reported from another study, underlining the levels of bicarbonate, FVC, FEV_1_ and BMI and nocturnal oxygen indices, as predictors of daytime hypercapnia in OSA patients [34]. Conversely, Kawata et al. [35], assessing OSA patients with daytime hypercapnia, found that hypercapnia was best predicted by AHI and less from BMI and vital capacity. In a study including COPD-only patients, hypercapnia associated with reduced lung function and ventilatory capability and specifically, with low pO_2_ and FEV_1_ and high residual volume, as well as low minute ventilation and high volumes of exhaled carbon dioxide [36]. In the study by Resta et al., who evaluated OS patients with and without hypercapnia separately, pO_2_ and FEV_1_ levels and body weight independently predicted the presence of pCO_2_ in OS patients, with pO_2_ being the best predictor. These findings coincide with our study results, highlighting the multifactorial genesis of hypercapnia in the group of patients with OS [25]. In summary, the current evidence, drawn mainly from OSA studies, as well as the findings from Resta et al. [12] and ours, shows that impaired respiratory mechanics and reduced ventilatory drive related to obesity, as well as more severe airflow obstruction, could explain the presence of hypercapnia in OS.

Certainly, our study is subject to some limitations. Firstly, this is a retrospective study including patients only from a single center. Nevertheless, our sleep laboratory is located at a tertiary university hospital in northeastern Greece and is the main center in this area, assessing a representative number of patients with suspected sleep disorders annually. Secondly, the present study has a retrospective design, and thus no causality could be established regarding the impact of hypercapnia on sleep and respiratory parameters in patients with OS. Thirdly, there was an overrepresentation of males in comparison to females in our study. This fact can be explained by the epidemiological aspects of both OSA and COPD, which show that both diseases are more frequently diagnosed in males than females [4,5]. Moreover, there was no recording of CO_2_ levels during sleep, which could have given valuable information in terms of nocturnal hypoventilation not only in hypercapnic but also in the normocapnic group. The latter group could have also presented with some degree of nocturnal hypoventilation before it becomes diurnal. Finally, there was a lack of information regarding the COPD related symptoms and specifically we could not show any differences in regard between hypercapnic and normocapnic patients with OS.

## 5. Conclusions

In conclusion, our results suggest that patients with OS and hypercapnia demonstrate increased BMI, more daytime sleepiness and exhibit worse respiratory function in wakefulness and sleep hypoxia characteristics compared with OS patients and normocapnia. Moreover, BMI and FVC were the major determinants of hypercapnia in our study. Further research is needed to better elucidate the impact of hypercapnia on outcomes of patients with OS.

## Figures and Tables

**Table 1:** Anthropometric characteristics between overlap syndrome patients with and without hypercapnia.

	Patients without Hypercapnia *n* = 108	Patients with Hypercapnia *n* = 55	*p*
Gender (males/females)	92/16	50/5	0.302
Age (years)	62 (51–68)	61 (55–68)	0.689
BMI (kg/m^2^)	35.2 (31.1–39.5)	39.7 (34.4–43.2)	0.001
Neck circumference (cm)	44 (41–47)	47 (44–50)	0.017
Waist circumference (cm)	124 (110–132)	132 (117.5–138)	0.013
Hip circumference (cm)	117 (110–123)	122 (110.5–129)	0.171
WHR	0.89 (0.79–0.95)	0.90 (0.81–1.03)	0.128
Former smokers (%)	81.5%	81.8%	0.357

Abbreviations: BMI: body mass index; WHR: waist-to-hip ratio.

**Table 2:** Comparison of sleep characteristics between overlap syndrome patients with and without hypercapnia.

	Patients without Hypercapnia *n* = 108	Patients with Hypercapnia *n* = 55	*p*
Recording time (min)	382 (360.3–402)	378.5 (359.5–396)	0.654
TST (min)	303 (248.3–338.5)	310 (264–354)	0.088
N1 (%)	20 (10.8–37.3)	12 (4–21)	0.001
N2 (%)	55.5 (42–70)	67 (50–83)	0.005
N3 (%)	6 (0–11.3)	7 (1–17)	0.204
REM (%)	8 (3–16)	6 (2–10)	0.237
AHI (events/hour)	39.5 (18–61)	41 (16–66.5)	0.403
ODI (events/hour)	35.5 (20.3–60.8)	63 (28–80)	0.004
Aver SpO_2_ (%)	91 (89–94)	89 (85.5–92)	<0.001
Min SpO_2_ (%)	77 (70–83)	68 (57–78)	<0.001
T < 90% (%)	20.7 (4–44.9)	38 (14.8–80.5)	0.006
Arousal index	30 (17–49.3)	21 (5.5–50)	0.118
Sleep efficiency (%)	79.3 (68.5–87.7)	85.5 (74.4–91.9)	0.033
ESS score	9 (6–12)	13 (7.5–17.5)	0.008

Abbreviations: AHI: apnea hypopnea index; Aver SpO_2_: average oxyhemoglobin saturation; ESS: Epworth sleepiness scale; Min SpO_2_: minimum oxyhemoglobin saturation; N1: sleep stage 1; N2: sleep stage 2; N3: sleep stage 3; ODI: oxygen desaturation index; REM: rapid eye movement; TST: total sleep time; T < 90%: time spent with oxyhemoglobin saturation <90%.

**Table 3:** Comparison of respiratory characteristics during wakefulness between overlap syndrome patients with and without hypercapnia.

	Patients without Hypercapnia *n* = 108	Patients with Hypercapnia *n* = 55	*p*
FEV_1_ (% predicted)	69.5 (58–79)	59 (45–75)	0.003
FVC (% predicted)	74.5 (62–91)	63 (54–82)	0.004
FEV_1_/FVC (%)	67 (62–69.1)	68 (63–69.6)	0.396
pH	7.43 (7.41–7.45)	7.41 (7.39–7.43)	0.001
pO_2_ (mmHg)	74 (66.3–82)	65 (58–71)	<0.001
pCO_2_ (mmHg)	41 (37.8–43)	49 (47–54)	<0.001

Abbreviations: FEV_1_: forced expiratory volume in first second; FVC: forced vital capacity; pCO_2_: carbon dioxide partial pressure; pO_2_, oxygen partial pressure.

## Data Availability

Data available on request from the authors.
